# Prolonged sitting reduces cerebral oxygenation in physically active young adults

**DOI:** 10.3389/fcogn.2024.1370064

**Published:** 2024-08-12

**Authors:** Brett D. Baker, Darla M. Castelli

**Affiliations:** ^1^Department of Kinesiology, Huston-Tillotson University, Austin, TX, United States; ^2^Department of Physical Therapy, Movement, and Rehabilitation Science, Northeastern University, Boston, MA, United States

**Keywords:** prolonged sitting, fNIRS, sedentary behavior, physical activity, young adults

## Abstract

**Introduction:**

Physical activity is known to enhance cognitive functioning across the lifespan, yet the effects of sedentary behaviors on cognitive functioning remain unclear. The purpose of this study was to determine how an acute daily bout of prolonged sitting influenced working memory, inhibitory control, and cerebral oxygenation (HbO_2_) in a sample of healthy young adults.

**Methods:**

Forty-one young adults (aged between 18–30 years of age) participated in an exploratory design intended to establish a control standard for determining how an acute 2-h bout of prolonged sitting influenced working memory, inhibitory control, and HbO_2_. The Flanker task, Simon task, and Delayed Match to Sample were utilized to assess inhibitory control and working memory, respectively, while functional near-infrared spectroscopy assessed HbO_2_. Participants were further subdivided into a physically active (Active) group and a physically inactive group (Inactive) based on self-reported physical activity participation. Paired sample *t*-tests were used to determine any changes in working memory, inhibitory control, and HbO_2_ from pre-to-post and between groups.

**Results:**

There were no differences in working memory or inhibitory control reaction time following prolonged sitting for the entire sample (*p* > 0.05) or between activity groups (*p* > 0.05). There was a significant reduction in Flanker accuracy post-prolonged sitting for both the congruent (*p* < 0.05) and incongruent (*p* < 0.05) conditions. For those in the Inactive group, there was no difference in HbO_2_ concentrations post-prolonged sitting. Those in the Active group exhibited a significant reduction in HbO_2_ during the Flanker Task following prolonged sitting (*p* < 0.05).

**Conclusions:**

An acute bout of daily prolonged sitting significantly reduced HbO_2_ in physically active young adults but not in inactive young adults. We recommend that future studies examining the effects of sedentary behaviors on microvasculature include an objective assessment of physical fitness and a direct measure of physical activity patterns and consider these values when assigning participants to the intervention condition.

## Introduction

The technological advances at the turn of the century have led to increased sedentary behavior (SB), defined as any waking behavior ≤ 1.5 METS done while in a sitting, reclining, or lying posture (Tremblay et al., 2017). Increased SB is due in part to increases in screen time (Sandercock et al., 2016; Sallis et al., 2020), as individuals mainly engage with digital screens while in a sitting or lying posture. In 2005, just 5% of adults and 7% of young adults (individuals aged 18–30) used one social media platform, but today, 79 and 90% report habitual use, respectively (Media Inquiries, 2023). The streaming video market has also seen a comparable increase in viewership, as there was an 842% increase between 2011 and 2020 (Netflix Revenue and Usage Statistics, 2023).

Lower energy expenditure resulting from more screen time and SB increases the risk of cardiovascular disease (CVD) (Biddle et al., 2017). Historically, CVD has been associated with later life; however, markers of CVD, such as high blood pressure, blood sugar, blood lipids, and excess abdominal fats, are currently on the rise, especially among young adults (Benjamin et al., 2018). Young adults under 30 now have CVD risk profiles similar to what was previously seen in older adults, partly because they are more sedentary and obese than their parents at the same age (Saydah et al., 2013).

In older adults, CVD risk factors are associated with cognitive dysfunction, higher risks of dementia (Schmidt et al., 2002), and poorer cognitive performance on inhibitory control tasks (Santos et al., 2017). Young adults with higher aerobic fitness outperform lower-fit individuals on executive function tasks (Liu et al., 2022; Loprinzi et al., 2023). The current context has raised concerns about SB's deleterious health outcomes and potential adverse effects on young adults' cognitive productivity.

Individuals between 18 and 30 are approaching or at their cognitive processing peak, marked by superior executive functions (Salthouse and Davis, 2006; Themanson et al., 2008). Executive functions are a group of higher-order cognitive processes that include planning, attention, inhibitory control, working memory, and cognitive flexibility (Chan et al., 2008) Executive functions are crucial in everyday life, as individuals constantly need to inhibit certain actions and store information for later use.

The young adult developmental stage of the lifespan and the effectiveness and efficiency of cognitive processing can mask the symptomology resulting from participation in risky behaviors (Åberg et al., 2009). However, according to cohort research by Salthouse (2009), age-related declines in reasoning and spatial orientation begin as early as the third decade of life (Salthouse, 2009). It is well-known that acute and chronic physical activity (PA) induces positive cognitive benefits, both functionally and structurally (Tomporowski, 2003; Colcombe et al., 2006; Ogoh and Ainslie, 2009; Voss et al., 2014). However, the acute and independent effects of prolonged sitting-based SB on cognitive processing and executive functions vary according to research methodology and study sample.

Using functional near-infrared spectroscopy (fNIRS) and the Stroop task to measure cerebral oxygenation (HbO_2_) and executive function respectively, Stoner et al. (2019) found that a 3 h bout of prolonged sitting based-SB did not change HbO_2_ or executive function performance. In contrast, Horiuchi et al. (2023) found that an acute bout of prolonged sitting significantly reduced Stroop task performance. Further, Perdomo et al. (2019) found no effect of acute prolonged sitting on cerebral blood flow velocity while Carter et al. (2018) found a significant reduction in cerebral blood flow velocity following an acute bout of prolonged sitting. The generalizability of these studies is limited by low sample sizes (*n* = 15 and 25, respectively), the use of middle-aged pre-hypertensive samples, and no control for the cognitive activity of the individuals during the prolonged sitting condition of the experiments. This study addresses these limitations by having a larger sample size, a healthy sample of young adults free of overt diseases and controls the cognitive environment of all individuals.

With the increasing evidence that SB is linked to CVD and CVD is linked to cognitive dysfunction, we must determine if prolonged sitting based-SB can attenuate brain function during a portion of the lifespan when we subjectively believe individuals are cognitively and physically healthy. Further, possible degradation of working memory prohibits the holding of information, and a lack of inhibitory control reduces workplace and school productivity. As such, the purpose of this study was to determine the effects of prolonged sitting on working memory, inhibitory control, and prefrontal cortex HbO_2_ to establish a control standard for a second study phase that assigned participants to an all-sitting-SB or intermittently PA interrupted sitting-SB. Only phase one is reported in this paper.

The following research questions guided this study:

- Do working memory and inhibitory control significantly change after a common daily bout of prolonged sitting among young adults? We anticipated that as little as 2 h of prolonged sitting would reduce working memory and inhibitory control. Specifically, the error rate would be higher and reaction time would be slower posttest than at baseline.
- Does the concentration of prefrontal cortex HbO_2_ significantly change after a common daily bout of prolonged sitting among young adults? It was anticipated that HbO_2_ would decline posttest compared to baseline.
- Are there differences in working memory, inhibitory control, and HbO_2_ concentrations due to a common daily bout of prolonged sitting by the amount of time individuals spent in PA during the week? We hypothesized that individuals who regularly exercise would have lower error rates, faster reaction times and elevated HbO_2_ following a bout of sitting when compared to individuals who do not regularly exercise.

## Materials and methods

### Participants

Once approved by the Institutional Review Board of the University of Texas at Austin, 41 young adults (29 female, 12 males; average age = 22 ± 3 years) were recruited through flyers and word of mouth. A power analysis computed a required sample size of 40 individuals (G^*^Power, v3.19.6). Individuals were excluded from participating if they were not between the ages of 18–30 or had self-reported CVD or cognitive impairment. Participants were given a $35 gift card for completing the study protocol. Informed consent was obtained from all participants in this study.

### Study design

This study was conducted in two phases: (a) part one was a pre/post exploratory design in which participants were exposed to 2 h of uninterrupted prolonged sitting to establish a control standard, and (b) participants were randomly assigned to 30 min of additional prolonged sitting-SB or 30 min of planned intermittent PA. The results for phase two are reported elsewhere while this study reports the results of part one.

Since sitting for 2 h is a common behavior among young adults (e.g., watching a movie, attending class), we wanted to quantify how an acute 2-h bout of prolonged sitting influenced working memory, inhibitory control, and HbO_2_ while controlling for caloric consumption, caffeine, and the environment. This study was completed across two study visits on back-to-back days. Study visit one was a familiarization visit, while study visit two was the primary data collection.

During familiarization, participants completed a consent form, a health history questionnaire, and the International Physical Activity Questionnaire (IPAQ) (Hagströmer et al., 2006). Brachial artery blood pressure was measured using an automated blood pressure cuff (Omron 3 Series, Model BP7100), and participants performed familiarization tests of working memory and inhibitory control; each test was completed four times to minimize the learning effect the next day.

During the experimental visit, participants came to the lab the morning after the familiarization visit following an overnight fast. At baseline, participants placed a FitBit (FitBit Sense) on their wrist to continuously monitor activity. They rated their subjective level of sleepiness and mental effort using the Karolinska Sleepiness Scale (Young et al., 2016) and the Paas Mental Effort Scale (Paas et al., 1994), respectively. Working memory, inhibitory control, and cerebral HbO_2_ were measured at rest to represent their baseline.

As directed by a registered dietician, the participants were given an organic meal replacement shake (Orgain Organic Nutritional Protein Shake) as breakfast. Each shake was 250 calories with macronutrient breakdown as follows: 8 g fat, 28 g carbs., and 16 g protein. Following, participants completed a 2-h bout of prolonged sitting. We recognize that bouts of prolonged sitting can range from 60 min to 12 h. The duration of sitting in this experiment was chosen to replicate a typical duration that young adults remain sedentary without significant energy expenditure, either in a classroom setting or while watching a movie or two episodes of a streaming video series. Participants sat in a recliner during these 2 h while watching episodes of Modern Marvels (History Channel); Modern Marvels was chosen as a non-stimulating history documentary. Importantly, all participants watched the same episodes in the same order. After 2 h of prolonged sitting, working memory, inhibitory control, and cerebral HbO_2_ were assessed.

### Physical activity

To ensure that participants were as sedentary as possible and to account for any postural change during the protocol, they wore FitBit Senses to assess movement.

### Alertness and mental effort

Participants subjectively rated their mental alertness and mental effort before and after prolonged sitting by using the Karolinska Sleepiness Scale and the Paas et al. (1994) subjective rating scale, respectively. Values for the Karolinska Sleepiness Scale range from 1 to 9 (e.g., 1 = extremely alert…9 = extremely sleepy), with lower values representing greater mental alertness, or less sleepiness. Values for the Paas subjective mental effort scale range from 1 to 9 (e.g., 1 = very, very low mental effort…9 = very, very high mental effort) with lower values representing lower levels of cognitive mental effort. These scales are valid and reliable (Paas et al., 1994; Kaida et al., 2006; Young et al., 2016) and were utilized to measure any changes in sleepiness and mood that could affect our outcome variables.

### Working memory and inhibitory control

Computer-based tests of working memory and inhibitory control were obtained from the Psychology Experiment Building Language (PEBL), an open-source software program. The cognitive tests were administered in a quiet indoor lab space, free of auditory and visual distractions.

We chose to utilize working memory and inhibitory control as our executive function measures as they are heavily researched and relevant functions. Every day, individuals need to consistently update and store information for future tasks and inhibit certain actions and behaviors to respond accurately and efficiently. We utilized two inhibitory control tests instead of measuring another executive function to measure the depth of a particular executive function rather than the breadth of all executive functions. For all cognitive tasks, accuracy and reaction time were the variables of interest.

The Delayed Match to Sample Task (DMS) measured working memory (Perez et al., 1987; Ahonen et al., 2012). In the DMS task, participants are shown one 4 × 4 matrix with yellow and red boxes. After 1 s, the matrix disappears, followed by a 6-s delay. After 6 s, two matrices appear, and participants must correctly select which matrix was previously displayed.

The Simon Task was used to measure inhibitory control, which requires individuals to suppress prepotent responses when presented with particular stimuli (Guiney and Machado, 2013). In the Simon task, participants are shown either a blue or a red circle on the screen. They are instructed to select the right shift key when the red circle appears and the left shift key when the blue circle appears.

The Flanker Task (Eriksen and Schultz, 1979) was also utilized to measure inhibitory control and requires individuals to specify a central stimulus's direction while ignoring other stimuli located on the periphery of the central stimulus. Participants were shown five arrowheads and asked to specify the central arrowhead's direction while pressing the left or right shift key.

### Prefrontal cortex oxygenation

**Figure 1:**
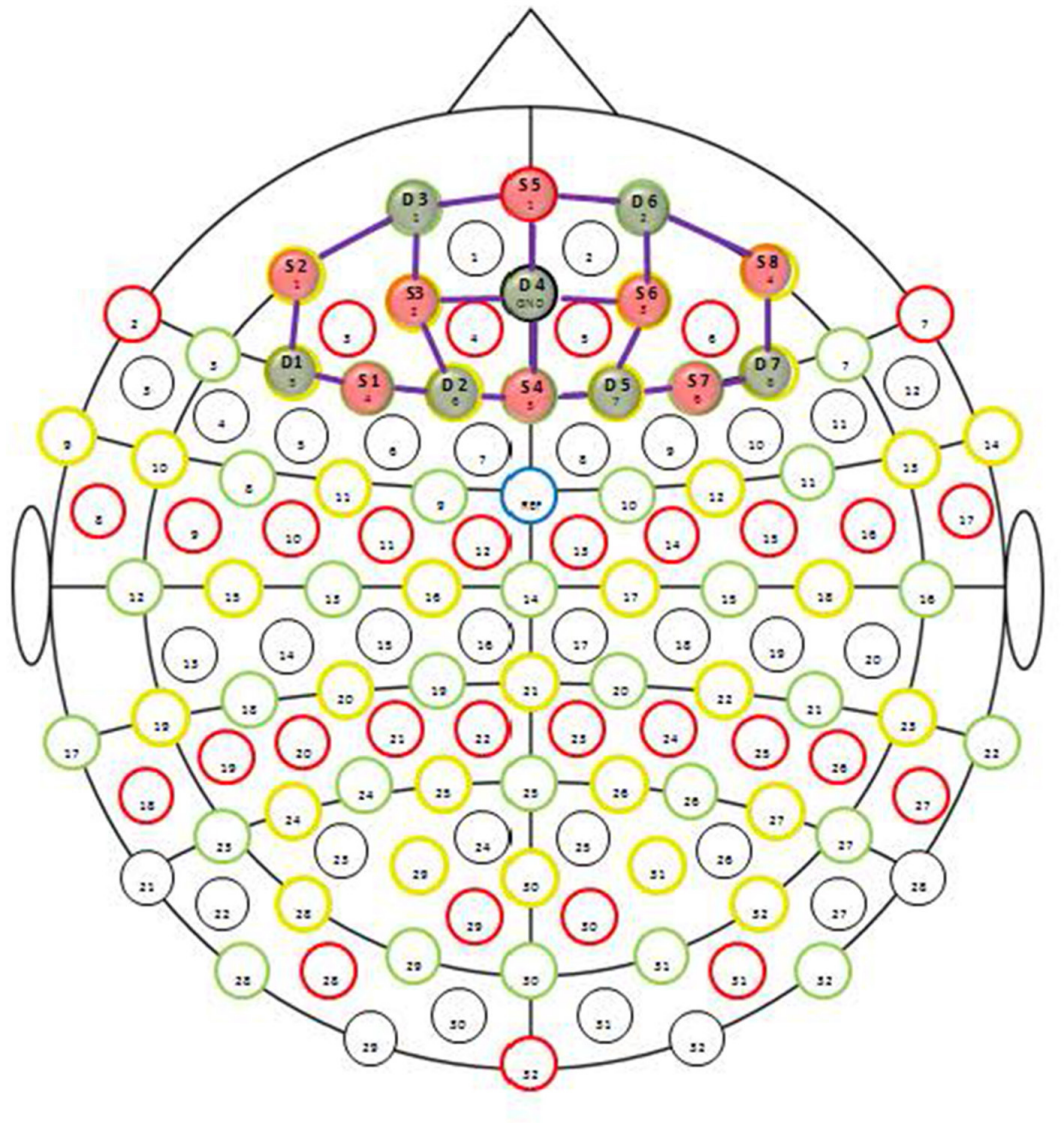
Prefrontal cortex fNIRS montage.

Functional near-infrared spectroscopy (fNIRS) (NIRScout; NIRx) was used to determine prefrontal cortex HbO_2_ concentrations during the working memory and inhibitory control tasks before and after prolonged sitting. We utilized a fNIRS system consisting of eight sources emitting light from two wavelengths, 760 and 850 nm, and seven detectors. The eight sources and seven detectors were placed on a fabric cap, like a swim cap, forming an array of 20 channels centered on the prefrontal cortex area (Figure 1). The prefrontal cortex was chosen as this brain region is known to be involved in executive functions. The head circumference of the participant determined the cap used. The optode for source five was placed at 10% of the nasion-inion distance with detectors 3 and 6 located adjacent. These positions correspond to the Fpz, Fp1 and Fp2, respectively, according to the international EEG 10-10 system. The overhead lights in the room were turned off to shield the optodes from ambient light.

Near-infrared light was emitted into the skull through the sources and was absorbed by hemoglobin chromophores, HbO_2_ and deoxygenated hemoglobin (HHb). As each chromophore has different absorption wavelengths, it was possible to determine the concentration of each chromophore from the volume of light absorbed. An indirect measure of neuronal activity, an increase in HbO_2_ and a corresponding decrease in HHb is used to describe an increase in cerebral oxygenation due to cortical activation (Obrig et al., 2000).

Data preprocessing for HbO_2_ concentrations was completed using the Homer3 package (v1.29.8) in MATLAB (v2017b) (Huppert et al., 2009). The data preprocessing steps were performed in the following order: (1) the raw signals were converted into optical densities using the hmrR_Intensity2OD function, (2) motion artifacts were detected using the hmrR_MotionArtifactByChannel function (time window set to 0.5 s, masked time range set to 3.0 s, standard deviation threshold set at 10, and amplitude threshold set at 5.0), (3) detected motion artifacts were corrected using the hmrR_MotionCorrectSplineSG function (parameter set at 0.99 with frame set at 10 s), (4) a bandpass filter was applied to the optical densities (set at 0.01–0.5 Hz), and (5) the artifact-corrected data were converted into micromolar changes of HbO_2_ using the hmrR_OD2Conc function (Obrig et al., 2000).

### Statistical analysis

All statistical analyses were conducted in SPSS (IBM SPSS Statistics, 29.0). Accuracy and reaction time data from the cognitive tests was transferred from PEBL to SPSS and HbO_2_ data from fNIRS were transferred from MATLAB to SPSS. Normality plots with tests were run to ensure all data were normally distributed. We used an alpha level of 0.05 for all statistical tests.

To confirm the fidelity of the treatment that participants were sedentary and were not ambulatory during the protocol, paired sample *t*-tests analyzed the step counts recorded before and after the acute bout of SB. To account for any changes in mental alertness and mental effort, paired sample t-tests were performed on participants' subjective rating of the Karolinska Sleepiness scale and the Paas mental effort scale, respectively.

To test the hypothesis that an acute 2-h bout of prolonged sitting significantly changed working memory and inhibitory control performance, paired sample *t*-tests were performed with reaction time and accuracy as the dependent variables.

To determine if an acute bout of prolonged sitting significantly affected cerebral oxygenation, the micromolar concentrations of HbO_2_ from each quality channel were analyzed by paired sample *t*-tests for each cognitive task.

To determine if there were differences in working memory, inhibitory control performance and HbO_2_ concentrations due to prolonged sitting by the amount of time individuals spent in PA during the week, we stratified all participants into one of two groups based on their time spent in moderate-to-vigorous physical activity (MVPA). The time participants spent in MVPA was determined by their responses to questions 22–25 on the IPAQ. Briefly, questions 22 & 23 address the frequency and time in which someone engaged in vigorous PA (e.g., aerobics, running, fast cycling) while questions 24 & 25 address the frequency and time in which someone engaged in moderate PA (e.g., regular cycling, swimming, doubles tennis). Individuals who met the MVPA guidelines over the past week were categorized into the Active group, while participants who did not were categorized into the Inactive group. Paired sample *t*-tests were performed to determine differences in working memory, inhibitory control, and HbO_2_ concentrations between groups.

## Results

There were 41 young adults who participated in this study. Complete participant characteristics can be seen in Table 1 while the characteristics for activity groups can be seen in Table 2.

**Table 1:** Participant characteristics.

**Variable**	**Mean±SD or *n* (%)**
Age, years	22 ± 3
**Sex**	
Female	29 (63)
Male	12 (27)
**Race**	
White	11 (27.5)
Black	2 (5)
Latino	8 (20)
Asian	18 (45)
Other	1 (2.5)
Education, years	15 ± 2
BMI	23.39 ± 5.11
Weekday sit minutes	442.1 ± 194.5
MVPA-min./week	129.1 ± 248.1

**Table 2:** Participant characteristics by activity group.

**Variable**	**Mean** ±**SD or** ***n*** **(%)**	
	**Inactive**	**Active**
Age, years	21 ± 3	22 ± 3
**Sex**	
Female	18 (75)	11 (64.71)
Male	6 (25)	6 (35.29)
**Race**	
White	2 (8.33)	9 (52.94)
Black	2 (8.33)	0 (0)
Latino	5 (20.83)	3 (17.65)
Asian	15 (62.50)	4 (23.53)^*^
Other	0 (0)	1 (5.88)
Education, years	15 ± 3	15 ± 1
BMI	24.40 ± 6.40	21.73 ± 1.68
Weekday sit minutes	460.00 ± 210.03	414.71 ± 179.50
MVPA-min./week	0.0 ± 0.0	311.20 ± 74.20^*^

^*^p-value < 0.05 between groups.

### Movement analysis, mental effort, and mental alertness

No differences in step count, mental alertness or mental effort were observed in the total sample or between activity groups (Table 3).

**Table 3:** Mean and standard deviation of step count, mental alertness, and mental effort.

	**Total sample**		**Active individuals**		**Inactive individuals**	
	**Pre**	**Post**	**Pre**	**Post**	**Pre**	**Post**
Step count	4.0 ± 6.5	4.6 ± 6.9	4.6 ± 7.6	5.5 ± 7.9	3.7 ± 5.8	4.0 ± 6.3
Mental alertness	4.8 ± 1.5	5.2± 1.9	5.0 ± 1.1	5.6 ± 1.9	4.7 ± 1.6	4.9 ± 1.9
Mental effort	3.7 ± 1.8	3.8 ± 1.6	3.8 ± 1.7	3.5 ± 1.2	3.7 ± 1.8	4.0 ± 1.8

### Working memory and inhibitory control

There were no differences in reaction time on any cognitive task either in the entire sample or by activity group (Table 4). There were no differences in accuracy performance on the Simon Task or Delayed Match to Sample task in the entire sample or between activity groups (Table 4). Accuracy performance on the Flanker Task was significantly worse than at baseline for both the Congruent and Incongruent conditions in the entire sample (Table 4).

**Table 4:** Mean and standard deviation of working memory and inhibitory control tests.

	**Total sample**		**Active individuals**		**Inactive individuals**	
**Reaction time (ms)**	**Pre**	**Post**	**Pre**	**Post**	**Pre**	**Post**
Flanker congruent	446.7 ± 41.3	447.5 ± 45.8	444.7 ± 45.4	440.0 ± 33.2	448.2 ± 39.1	453.0 ± 53.3
Flanker incongruent	481.1 ± 39.3	484.2 ± 50.1	477.2 ± 42.1	480.7 ± 50.9	484.0 ± 37.8	486.8 ± 50.4
Simon congruent	400.7 ± 42.5	402.4 ± 44.1	391.2 ± 42.3	400.0 ± 43.0	407.4 ± 42.2	404.0 ± 45.8
Simon incongruent	430.4 ± 42.6	427.4 ± 56	424.3 ± 51.8	430.7 ± 60.5	435.3 ± 34	424.7 ± 53.4
Delayed match to sample	1355.3 ± 445.1	1325.5 ± 354.3	1380.7 ± 565.7	1257.2 ± 386.4	1337.3 ± 347.9	1374.0 ± 329.4
**Accuracy (%correct)**	**Pre**	**Post**	**Pre**	**Post**	**Pre**	**Post**
Flanker congruent	98.9 ± 2.4	97.7 ± 3.5^*^	98.2 ± 2.9	97.8 ± 2.3	99.4 ± 1.7	97.8 ± 4.1
Flanker incongruent	96.0 ± 3.5	94.6 ± 5.4^*^	96.1 ± 4.1	93.7 ± 5.3	96.3 ± 3.0	94.8 ± 5.9
Simon congruent	98.3 ± 2.7	97.8 ± 3.5	97.4 ± 3.0	97.6 ± 3.3	98.8 ± 2.4	97.9 ± 3.8
Simon incongruent	93.8 ± 4.8	94.9 ± 4.8	93.5 ± 4.8	94.7 ± 4.6	94.0 ± 4.9	95.0 ± 5.1
Delayed match to sample	93.2 ± 5.1	93.4 ± 6.7	92.4 ± 5.3	92.9 ± 6.6	93.8 ± 4.9	93.8 ± 7.0

^*^p-value < 0.05 pre compared to post.

### Prefrontal cortex oxygenation

**Figure 2:**
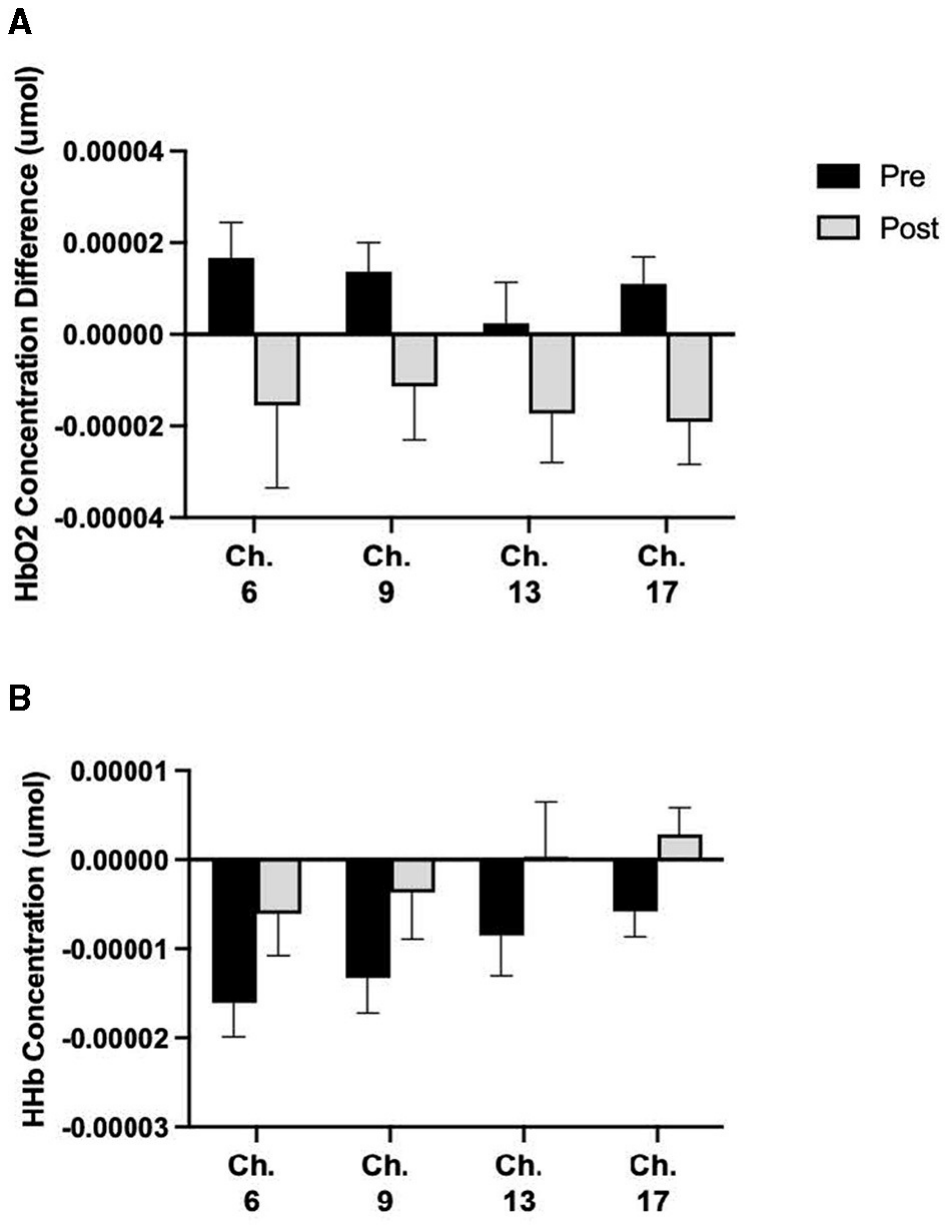
Total sample differences in HbO_2_ concentrations. Means ± SE showing **(A)** significant HbO_2_ concentration changes during the Flanker Task before and after 2 h of SB and **(B)** significant HHb concentration changes during the Flanker Task before and after 2 h of SB. From left to right, Cohen's *d* effect sizes for **(A)**: 0.32, 0.41, 0.31, 0.42 and **(B)**: 0.42, 0.41, 0.33, 0.59.

The analysis included 18 channels, with two channels eliminated due to poor signal quality. There was no difference in HbO_2_ values during the Simon or DMS task following prolonged sitting for the entire sample (*p* > 0.05) or between activity groups (*p* > 0.05). Paired sample *t*-tests revealed a significant reduction in cerebral oxygenation for 4 channels during the Flanker task following prolonged sitting (Figure 2).

**Figure 3:**
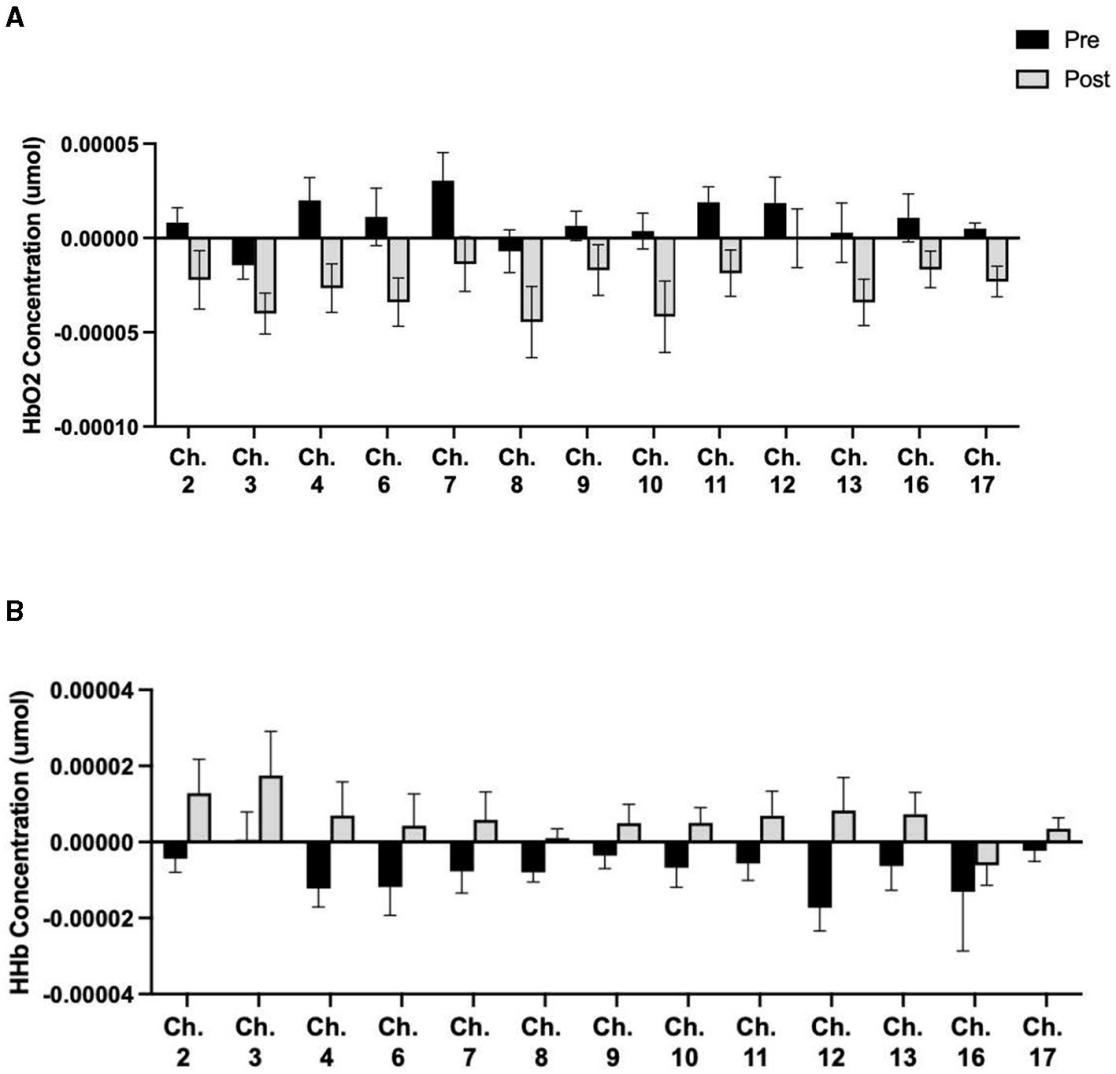
Active group differences in HbO_2_. Means ± SE showing **(A)** significant HbO_2_ concentration changes during the Flanker Task before and after 2 h of SB and **(B)** significant HHb concentration changes during the Flanker Task before and after 2 h of SB. From left to right, Cohen's *d* effect sizes for **(A)**: 0.56, 0.64, 0.80, 0.69, 1.19, 0.68, 0.47, 0.62, 0.82, 0.63, 0.73, 0.62, 1.18 and **(B)**: 0.69, 0.69, 0.59, 0.74, 0.75, 0.83, 0.51, 0.56, 0.50, 0.94, 0.56, 0.62, 0.84.

**Figure 4:**
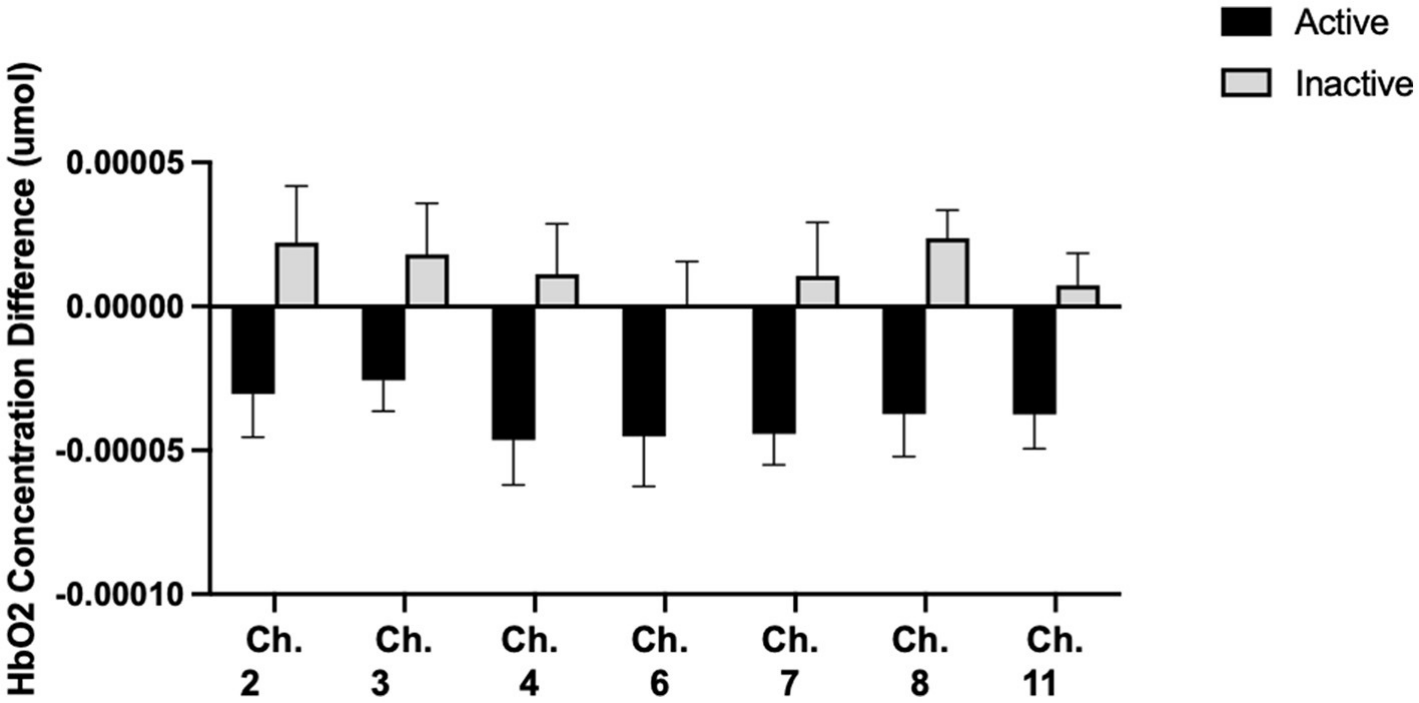
Group differences in HbO_2_ concentrations. Means ± SE showing significant HbO_2_ concentration differences between activity groups before and after 2 h of SB. From left to right, Cohen's *d* effect sizes are: 0.71, 0.66, 0.85, 0.70, 0.80, 1.27, 0.98.

Additionally, there were differences in HbO_2_ concentrations following prolonged sitting by activity group. For the Inactive group, there was no difference in HbO_2_ concentrations for any channel during any task. However, for the Active group, there was a significant reduction in cerebral oxygenation for 13 channels during the Flanker Task (Figure 3). There were also differences in HbO_2_ between activity groups. Compared to the Inactive group, the Active group exhibited significantly lower HbO_2_ values following 2 h of prolonged sitting than the Inactive group in seven channels (*p* < 0.05) (Figure 4).

## Discussion

The present study aimed to determine how an acute bout of prolonged sitting based-SB influenced working memory, inhibitory control and HbO_2_ in a population of young adults. This exploratory study would then allow us to identify potential strata for randomization assignment into different conditions in the second phase of our study. We feel the findings are worth sharing given the potential for confounding effects for future studies.

We hypothesized that an acute bout of prolonged sitting would reduce executive function performance and HbO_2_; however, the dose of 2 h of sitting—which we chose to replicate the typical duration of time that undergraduates remain seated in class or at home—in the recliner did not result in the anticipated losses in reaction time, but did result in the anticipated reductions of Flanker accuracy and HbO_2_ during the Flanker Task. Also, contrary to our original hypothesis, physically active individuals did not demonstrate elevated HbO_2_ following 2 h of prolonged sitting but exhibited a significant reduction in HbO_2_.

The lack of a slowed reaction time on the cognitive tasks and a detrimental decrease in DMS and Simon task accuracy likely occurred for three reasons: (a) this was a healthy sample of young adults who are used to sitting for 2 h at a time, (b) the DMS did not produce enough variability in the responses (most participants did well), (c) the Simon Task may be an easier inhibitory control test than the Flanker Task due to design of the Simon Task in which there are no “distractors” or “flankers” as in the Flanker Task. Healthy young adults are at their cognitive peak and will likely respond faster or more accurately at no other time. In hindsight, using a 2 or 3 n-back task and the Stroop Task might have been more demanding and likely would have produced more significant score variability than the DMS and Simon Task we chose. Although we anticipated that the behaviors might be significantly different after 2 h of sitting, the sedentary time is equivalent to the screen time of watching a movie or multiple episodes of a TV show. In this age group, individuals may have unknowingly trained themselves to adapt to 2 h of sitting without any negative cognitive ramifications. Only two other studies have investigated the acute effects of prolonged sitting on executive function in young adults (Stoner et al., 2019; Horiuchi et al., 2023). Both studies used a 3-h bout of prolonged sitting to determine changes in the Stroop task and Trail-Making Test. Stoner et al. (2019) found no change in Stroop Task performance following prolonged sitting while Horiuchi et al. (2023) found a significant reduction in both Stroop task and Trail-Making Test performance.

In line with our hypothesis, HbO_2_ was significantly reduced during the Flanker task following prolonged sitting. This finding is similar to that of Carter et al. (2018) and Perdomo et al. (2019), who observed significant reductions in cerebral blood flow velocity following acute bouts of sitting. However, their participants were middle-aged (Carter et al., 2018) and pre-hypertensive individuals (Perdomo et al., 2019), whereas our population was healthy young adults without any reported incidences of disease. In addition, Carter et al. (2018) and Perdomo et al. (2019) had individuals sit uninterrupted for 3 h and 40 min and 4 h, respectively. In our study, individuals sat uninterrupted for 2 h, thus signifying that an acute bout of sitting of only 2 h is enough to significantly affect the cerebral circulation in young adults.

Our study took place in the morning, and the importance of breakfast on cognition must be mentioned. As glucose is the main energy source for the brain, carbohydrate consumption is suspected to result in short-term improvements in cognitive functioning. Further, breakfast reduces cognitive decline throughout the morning when compared to fasting or skipping breakfast (Pollitt, 1995; Liu et al., 2013). Related, it is thought that the post-prandial declines in glucose after 2–3 h of ingestion could lead to cognitive decline as noted in our study (Cooper et al., 2012). However, a recent systematic review (Philippou and Constantinou, 2014) and a randomized crossover trial (Marchand et al., 2020) found mixed reviews and no effect of postprandial glycemia on cognition, respectively. As each individual in our study had a unique breakfast routine, it is unknown how consuming a meal replacement shake in place of that may have influenced their metabolism and thus cognition.

### Sitting-induced vascular dysfunction and cognitive functioning

Researchers have recently demonstrated that acute bouts of prolonged sitting lasting between 1 and 3 h significantly increase vascular dysfunction measured by decreased lower extremity shear stress and blood flow (Ballard et al., 2017; Morishima et al., 2017; Climie et al., 2018; Credeur et al., 2019). Although not measured, we hypothesize that calf blood pooling and the subsequent decreased venous return, decreased cardiac output, and decreases in shear stress-induced blood flow contributed to the significant decline in HbO_2_ found in our study and the decline in cerebral blood flow velocity found in others (Carter et al., 2018; Perdomo et al., 2019). In our study we controlled for lower limb movements by ensuring all participants did not move from the recliner and encouraged participants to refrain from fidgeting by keeping feet flat on the floor.

We believed a decline in HbO_2_ would negatively affect working memory and inhibitory control. If HbO_2_ declines, the amount of neuronal resources available for cognitive tasks and demands is decreased due to the decline in oxygen. Yet, the significant reduction in HbO_2_ during the Flanker task was not associated with the significant decrease in Flanker accuracy. As there were no changes in subjective measures of sleepiness and mental effort by the participants, we presume the attention levels of participants decreased due to the watching of documentaries, although we have no objective measure for this. Only two other studies have also investigated the effects of an acute bout of sitting on both HbO_2_ and executive function. However, Stoner et al. (2019) found no differences on Stroop Task performance following 3 h of prolonged sitting, while Horiuchi et al. (2023) found significant reductions in Stroop Task performance.

Contrary to our hypothesis, physically active individuals had attenuated HbO_2_ following an acute bout of prolonged sitting; physically inactive individuals did not experience any changes in HbO_2_. As aerobic exercise is known to increase vascular health (Spence et al., 2013; Groot et al., 2016), our results—that the active individuals had a decline in HbO_2_–are noteworthy. Prolonged sitting has been shown to significantly reduce lower extremity blood flow in both active and inactive young adults (Garten et al., 2019; Morishima et al., 2020; Liu et al., 2021). Liu et al. (2021) noticed a significant difference in the extent of reduction of lower limb blood flow between groups. Although an acute bout of prolonged sitting significantly reduced lower limb blood flow in both groups, as the active group had a significantly larger lower limb blood flow at baseline, they had a greater capacity to decrease after sitting (Liu et al., 2021). We speculate something similar occurred between our Active and Inactive groups as well. As physical activity is known to increase lower limb blood flow, our Active individuals had a greater capacity, or a higher ceiling, for lower limb blood flow to diminish compared to Inactive individuals. Although not measured, lower limb blood flow would be reduced to a greater extent in the Active group due to their higher baseline values. We hypothesize that the importance of the reduction of lower limb blood flow in this group due to venous pooling in the lower extremities allowed for a significant reduction in HbO_2_, which was not seen in the Inactive group.

## Limitations and strengths

Our study is not without limitations. First, the DMS and Simon Task may not have been difficult enough to elicit any variation in scores following prolonged sitting. Second, we did not have any measure for lower limb blood flow and shear stress which we hypothesize is responsible for the changes we observed in HbO_2_. Third, we used a self-report MVPA measure to identify our activity groups rather than an objective assessment of fitness, such as VO_2_max. Fourth, female participants' menstrual cycle was not controlled which may affect vascular function and cerebral blood flow. And fifth, we realize this study's findings are only generalizable to other similar indoor prolonged sitting-based SBs.

Only two other studies have investigated how an acute bout of sitting influences both executive function and HbO_2_ in young adults (Stoner et al., 2019; Horiuchi et al., 2023). However, their fNIRS device only included one optode. Comparatively, the strengths of this study are numerous. We had a larger sample size (*n* = 41), measured HbO_2_ across fifteen optodes, and ensured that each individual had the same cognitive workload. To the best of our knowledge, ours is the first study to investigate the differential effects an acute bout of prolonged sitting has on physically active and physically inactive individuals.

## Conclusions

An acute bout of daily prolonged sitting significantly reduced HbO_2_ in active young adults but not in inactive young adults. Future studies should (1) determine if prolonged sitting affects active and inactive individuals differently using an objective assessment of fitness, (2) employ more cognitively demanding tasks, and (3) investigate how an acute bout of prolonged sitting affects both peripheral and cerebral microvasculature function through lower limb blood flow and cerebral HbO_2_, respectively.

## Data availability statement

The original contributions presented in the study are included in the article/Supplementary material, further inquiries can be directed to the corresponding author.

## Ethics statement

The studies involving humans were approved by the University of Texas at Austin. The studies were conducted in accordance with the local legislation and institutional requirements. The participants provided their written informed consent to participate in this study.

## Author contributions

BB: Conceptualization, Methodology, Project administration, Validation, Data curation, Formal analysis, Investigation, Visualization, Writing – original draft. DC: Conceptualization, Methodology, Project administration, Validation, Resources, Supervision, Writing – review & editing.

## References

[B1] Åberg M. A. I. Pedersen N. L. Torén K. Svartengren M. Bäckstrand B. Johnsson T.. (2009). Cardiovascular fitness is associated with cognition in young adulthood. Proc. Nat. Acad. Sci. U. S. A. 106, 20906–20911. 10.1073/pnas.0905307106 19948959 PMC2785721

[B2] Ahonen B. Dunham C. Getty E. Kosmowski K. J. (2012). The effects of time of day and practice on cognitive abilities: the PEBL pursuit rotor, compensatory tracking, match-to-sample, and TOAV tasks. PEBL Techn. Rep. Ser. 2, 1–5.

[B3] Ballard K. D. Duguid R. M. Berry C. W. Dey P. Bruno R. S. Ward R. M.. (2017). Effects of prior aerobic exercise on sitting-induced vascular dysfunction in healthy men. Eur. J. Appl. Physiol. 117, 2509–2518. 10.1007/s00421-017-3738-2 29018989

[B4] Benjamin E. J. Virani S. S. Callaway C. W. Chamberlain A. M. Chang A. R. Cheng S.. (2018). Heart disease and stroke statistics-−2018 update: a report From the American Heart Association. Circulation 137, e67–492. 10.1161/CIR.0000000000000558 29386200

[B5] Biddle S. J. H. García Bengoechea E. Pedisic Z. Bennie J. Vergeer I. Wiesner G. (2017). Screen time, other sedentary behaviours, and obesity risk in adults: a review of reviews. Curr. Obes. Rep. 6, 134–147. 10.1007/s13679-017-0256-9 28421472

[B6] Carter S. E. Draijer R. Holder S. M. Brown L. Thijssen D. H. J. Hopkins N. D. (2018). Regular walking breaks prevent the decline in cerebral blood flow associated with prolonged sitting. J. Appl. Physiol. 125, 790–798. 10.1152/japplphysiol.00310.2018 29878870

[B7] Chan R. Shum D. Toulopoulou T. Chen E. (2008). Assessment of executive functions: review of instruments and identification of critical issues. Arch. Clin. Neuropsychol. 23, 201–216. 10.1016/j.acn.2007.08.010 18096360

[B8] Climie R. E. Wheeler M. J. Grace M. Lambert E. A. Cohen N. Owen N.. (2018). Simple intermittent resistance activity mitigates the detrimental effect of prolonged unbroken sitting on arterial function in overweight and obese adults. J. Appl. Physiol. 125, 1787–1794. 10.1152/japplphysiol.00544.2018 30188800

[B9] Colcombe S. J. Erickson K. I. Scalf P. E. Kim J. S. Prakash R. McAuley E.. (2006). Aerobic exercise training increases brain volume in aging humans. J. Gerontol. Ser. A 61, 1166–1170. 10.1093/gerona/61.11.1166 17167157

[B10] Cooper S. B. Bandelow S. Nute M. L. Morris J. G. Nevill M. E. (2012). Breakfast glycaemic index and cognitive function in adolescent school children. Br. J. Nutr. 107, 1823–1832. 10.1017/S0007114511005022 22017815

[B11] Credeur D. P. Miller S. M. Jones R. Stoner L. Dolbow D. R. Fryer S. M.. (2019). Impact of prolonged sitting on peripheral and central vascular health. Am. J. Cardiol. 123, 260–266. 10.1016/j.amjcard.2018.10.014 30409414

[B12] Eriksen C. W. Schultz D. W. (1979). Information processing in visual search: a continuous flow conception and experimental results. Percept. Psychophys. 25, 249–263. 10.3758/BF03198804 461085

[B13] Garten R. S. Hogwood A. C. Weggen J. B. Fralin R. C. LaRosa K. Lee D.. (2019). Aerobic training status does not attenuate prolonged sitting-induced lower limb vascular dysfunction. Appl. Physiol. Nutr. Metab. 44, 425–433. 10.1139/apnm-2018-0420 30257099

[B14] Groot H. J. Rossman M. J. Garten R. S. Wang E. Hoff J. Helgerud J.. (2016). The effect of physical activity on passive leg movement-induced vasodilation with age. Med. Sci. Sports Exerc. 48, 1548–1557. 10.1249/MSS.0000000000000936 27031748 PMC4949157

[B15] Guiney H. Machado L. (2013). Benefits of regular aerobic exercise for executive functioning in healthy populations. Psychon. Bull. Rev. 20, 73–86. 10.3758/s13423-012-0345-4 23229442

[B16] Hagströmer M. Oja P. Sjöström M. (2006). The International Physical Activity Questionnaire (IPAQ): a study of concurrent and construct validity. Public Health Nutr. 9, 755–762. 10.1079/PHN2005898 16925881

[B17] Horiuchi M. Pomeroy A. Horiuchi Y. Stone K. Stoner L. (2023). Effects of intermittent exercise during prolonged sitting on executive function, cerebrovascular, and psychological response: a randomized crossover trial. J. Appl. Physiol. 135, 1421–1430. 10.1152/japplphysiol.00437.2023 37942532 PMC12088687

[B18] Huppert T. J. Diamond S. G. Franceschini M. A. Boas D. A. (2009). HomER: a review of time-series analysis methods for near-infrared spectroscopy of the brain. Appl. Opt. 48, D280–D298. 10.1364/AO.48.00D280 19340120 PMC2761652

[B19] Kaida K. Takahashi M. Akerstedt T. Nakata A. Otsuka Y. Haratani T.. (2006). Validation of the Karolinska Sleepiness Scale against performance and EEG variables. Clin. Neurophysiol. 117, 1574–1581. 10.1016/j.clinph.2006.03.011 16679057

[B20] Liu H. O'Brien M. W. Johns J. A. Kimmerly D. S. (2021). Does aerobic fitness impact prolonged sitting-induced popliteal artery endothelial dysfunction? Eur. J. Appl. Physiol. 121, 3233–3241. 10.1007/s00421-021-04796-0 34417882

[B21] Liu J. Hwang W.-T. Dickerman B. Compher C. (2013). Regular breakfast consumption is associated with increased IQ in kindergarten children. Early Hum. Dev. 89, 257–262. 10.1016/j.earlhumdev.2013.01.006 23395328 PMC3606659

[B22] Liu Y. Zhu L. Cai K. Dong X. Xiong X. Liu Z.. (2022). Relationship between cardiorespiratory fitness and executive function in young adults: mediating effects of gray matter volume. Brain Sci. 12:1441. 10.3390/brainsci12111441 36358366 PMC9688695

[B23] Loprinzi P. D. Roig M. Tomporowski P. D. Javadi A.-H. Kelemen W. L. (2023). Effects of acute exercise on memory: considerations of exercise intensity, post-exercise recovery period and aerobic endurance. Mem. Cognit. 51, 1011–1026. 10.3758/s13421-022-01373-4 36401115 PMC9676734

[B24] Marchand O. M. Kendall F. E. Rapsey C. M. Haszard J. J. Venn B. J. (2020). The effect of postprandial glycaemia on cognitive function: a randomised crossover trial. Br. J. Nutr. 123, 1357–1364. 10.1017/S0007114520000458 32046793

[B25] Media Inquiries (2023). Social Media Fact Sheet. Pew Research Center: Internet, Science and Tech (blog). https://www.pewresearch.org/internet/fact-sheet/social-media/ (accessed July 25, 2023).

[B26] Morishima T. Restaino R. M. Walsh L. K. Kanaley J. A. Padilla J. (2017). Prior exercise and standing as strategies to circumvent sitting-induced leg endothelial dysfunction. Clin. Sci. 131, 1045–1053. 10.1042/CS20170031 28385735 PMC5516793

[B27] Morishima T. Tsuchiya Y. Ueda H. Tsuji K. Ochi E. (2020). Sitting-induced endothelial dysfunction is prevented in endurance-trained individuals. Med. Sci. Sports Exerc. 52:1. 10.1249/MSS.0000000000002302 32079922

[B28] Netflix Revenue and Usage Statistics (2023). Business of Apps. Available at: https://www.businessofapps.com/data/netflix-statistics/ (accessed July 25, 2023).

[B29] Obrig H. Wenzel R. Kohl M. Horst S. Wobst P. Steinbrink J.. (2000). Near-infrared spectroscopy: does it function in functional activation studies of the adult brain? Int. J. Psychophysiol. 35, 125–142. 10.1016/S0167-8760(99)00048-3 10677642

[B30] Ogoh S. Ainslie P. N. (2009). Cerebral blood flow during exercise: mechanisms of regulation. J. Appl. Physiol. 107, 1370–1380. 10.1152/japplphysiol.00573.2009 19729591

[B31] Paas F. G. Van Merriënboer J. J. Adam J. J. (1994). Measurement of cognitive load in instructional research. Percept. Mot. Skills 79, 419–430. 10.2466/pms.1994.79.1.419 7808878

[B32] Perdomo S. J. Gibbs B. B. Kowalsky R. J. Taormina J. M. Balzer J. R. (2019). Effects of alternating standing and sitting compared to prolonged sitting on cerebrovascular hemodynamics. Sport Sci. Health 15, 375–383. 10.1007/s11332-019-00526-4 31814853 PMC6897374

[B33] Perez W. A. Masline P. J. Ramsey E. G. Urban K. E. (1987). Unified Tri-services Cognitive Performance Assessment Battery: Review and Methodology (U). San Antonio, TX: Armstrong Aerospace Medical Research Laboratory, DTIC Document ADA181697.

[B34] Philippou E. Constantinou M. (2014). The influence of glycemic index on cognitive functioning: a systematic review of the evidence. Adv. Nutr. 5, 119–130. 10.3945/an.113.004960 24618754 PMC3951795

[B35] Pollitt E. (1995). Does breakfast make a difference in school? J. Am. Diet. Assoc. 95, 1134–1139. 10.1016/S0002-8223(95)00306-1 7560685

[B36] Sallis J. F. Conway T. L. Cain K. L. Geremia C. Bonilla E. Spoon C. (2020). Electronic devices as correlates of sedentary behavior and screen time among diverse low-income adolescents during the school year and summer time. J. Heal. Eat. Act. Living 1, 27–40. 10.51250/jheal.v1i1.7 37790138 PMC10544927

[B37] Salthouse T. Davis H. (2006). Organization of cognitive abilities and neuropsychological variables across the lifespan. Dev. Rev. 26, 31–54. 10.1016/j.dr.2005.09.001

[B38] Salthouse T. A. (2009). When does age-related cognitive decline begin? Neurobiol. Aging 30, 507–514. 10.1016/j.neurobiolaging.2008.09.023 19231028 PMC2683339

[B39] Sandercock G. R. H. Alibrahim M. Bellamy M. (2016). Media device ownership and media use: associations with sedentary time, physical activity and fitness in english youth. Prev. Med. Rep. 4, 162–168. 10.1016/j.pmedr.2016.05.013 27413678 PMC4929126

[B40] Santos P. P. Da Silveira P. S. Souza-Duran F. L. Tamashiro-Duran J. H. Scazufca M. Menezes P. R.. (2017). Prefrontal-parietal white matter volumes in healthy elderlies are decreased in proportion to the degree of cardiovascular risk and related to inhibitory control deficits. Front. Psychol. 8:57. 10.3389/fpsyg.2017.00057 28184203 PMC5266720

[B41] Saydah S. Bullard K. M. Imperatore G. Geiss L. Gregg E. W. (2013). Cardiometabolic risk factors among US adolescents and young adults and risk of early mortality. Pediatrics 131, e679–e686. 10.1542/peds.2012-2583 23420920 PMC4560449

[B42] Schmidt R. Schmidt H. Curb J. D. Masaki K. White L. R. Launer L. J. (2002). Early inflammation and dementia: a 25-year follow-up of the Honolulu-Asia aging study. Ann. Neurol. 52, 168–174. 10.1002/ana.10265 12210786

[B43] Spence A. L. Carter H. H. Naylor L. H. Green D. J. (2013). A prospective randomized longitudinal study involving 6 months of endurance or resistance exercise. Conduit Artery Adaptation in Humans. J. Physiol. 591, 1265–1275. 10.1113/jphysiol.2012.247387 23247114 PMC3607870

[B44] Stoner L. Willey Q. Evans W. S. Burnet K. Credeur D. P. Fryer S.. (2019). Effects of acute prolonged sitting on cerebral perfusion and executive function in young adults: a randomized cross-over trial. Psychophysiology 56:e13457. 10.1111/psyp.13457 31420883

[B45] Themanson J. R. Pontifex M. B. Hillman C. H. (2008). Fitness and action monitoring: evidence for improved cognitive flexibility in young adults. Neuroscience 157, 319–328. 10.1016/j.neuroscience.2008.09.014 18845227 PMC2657808

[B46] Tomporowski P. D. (2003). Effects of acute bouts of exercise on cognition. Acta Psychol. 112, 297–324. 10.1016/S0001-6918(02)00134-8 12595152

[B47] Tremblay M. S. Aubert S. Barnes J. D. Saunders T. J. Carson V. Latimer-Cheung A. E.. (2017). Sedentary Behavior Research Network (SBRN) – Terminology Consensus Project Process and Outcome. Int. J. Behav. Nutr. Phys. Act. 14:75. 10.1186/s12966-017-0525-8 28599680 PMC5466781

[B48] Voss M. W. Carr L. J. Clark R. Weng T. (2014). Revenge of the ‘sit' ii: does lifestyle impact neuronal and cognitive health through distinct mechanisms associated with sedentary behavior and physical activity? Ment. Health Phys. Act. 7, 9–24. 10.1016/j.mhpa.2014.01.001

[B49] Young J. Q. Boscardin C. K. van Dijk S. M. Abdullah R. Irby D. M. Sewell J. L.. (2016). Performance of a cognitive load inventory during simulated handoffs: evidence for validity. SAGE Open Med. 4:2050312116682254. 10.1177/2050312116682254 28348737 PMC5354177

