# Supplementary material for: Prolonged sitting reduces cerebral oxygenation in physically active young adults
Source: Front Cognit. 2024 Aug 12;3:1370064. doi: 10.3389/fcogn.2024.1370064 (PMC13281053; doi:10.3389/fcogn.2024.1370064)
Supplement: Supplementary file 1 [file Data_Sheet_1.PDF]

Supplementary Table. SB-induced changes in HbO2 and HHB for the Active group

Channel	HbO2				HHb			
	Pre	Post	*p*	Cohen's D	Pre	Post	*p*	Cohen's D
1	-0.074E-05	-2.158E-05	0.24	0.202	7.405E-06	-3.151E-06	0.06	0.588
2	0.818E-05	-2.221E-05	0.033*	0.56	-4.389E-06	12.85E-06	0.047*	-0.685
3	-1.44E-05	-4.01E-05	0.016*	0.642	0.656E-06	17.525E-06	0.029*	-0.685
4	1.995E-05	-2.654E-05	0.005*	0.798	-12.240E-06	6.945E-06	0.039*	-0.593
6	1.111E-05	-3.402E-05	0.011*	0.692	-11.182E-06	4.271E-06	0.049*	-0.742
7	3.045E-05	-1.385E-05	<0.001*	1.192	-7.724E-06	5.851E-06	0.047*	-0.753
8	-0.705E-05	-4.454E-05	0.012*	0.684	-7.984E-06	1.052E-06	0.01*	-0.826
9	0.643E-05	-1.701E-05	0.04*	0.468	-3.578E-06	4.951E-06	0.045*	-0.511
10	0.368E-05	-4.175E-05	0.019*	0.616	-6.745E-06	5.064E-06	0.032*	-0.564
11	1.897E-05	-1.863E-05	0.003*	0.822	-5.624E-06	6.896E-06	0.031*	-0.504
12	1.860E-05	-0.009E-05	0.012*	0.627	-17.290E-06	8.268E-06	0.034*	-0.943
13	0.284E-05	-3.417E-05	0.007*	0.73	-6.32E-06	7.306E-06	0.041*	-0.585
14	1.159E-05	-1.202E-05	0.068	0.465	-1.915E-06	-0.957E-06	0.447	-0.038
16	1.073E-05	-1.664E-05	0.033*	0.624	-9.011E-06	3.073E-06	0.04*	-0.623
17	0.499E-05	-2.303E-05	0.002*	1.182	-2.281E-06	3.517E-06	0.034*	-0.836
18	-0.579E-05	-3.596E-05	0.102	0.39	7.259E-06	-1.805E-06	0.07	0.66
19	-0.669E-05	-1.739E-05	0.228	0.191	0.545E-06	7.2141E-06	0.178	-0.246
20	-1.724E-05	-2.772E-05	0.368	0.092	6.5783E-06	12.042E-06	0.268	-0.184

*p-value < 0.05 between pre and post
